# Dynamic alterations in monocyte numbers, subset frequencies and activation markers in acute and convalescent COVID-19 individuals

**DOI:** 10.1038/s41598-021-99705-y

**Published:** 2021-10-12

**Authors:** Anuradha Rajamanickam, Nathella Pavan Kumar, Arul Nancy Pandiarajan, Nandhini Selvaraj, Saravanan Munisankar, Rachel Mariam Renji, Vijayalakshmi Venkatramani, Manoj Murhekar, Jeromie W. V. Thangaraj, Muthusamy Santhosh Kumar, C. P. Girish Kumar, Tarun Bhatnagar, Manickam Ponnaiah, R. Sabarinathan, V. Saravanakumar, Subash Babu

**Affiliations:** 1 International Center for Excellence in Research - ICMR- National Institute for Research in Tuberculosis, Chennai, TamilNadu India; 2 Immunology-ICMR-National Institute for Research in Tuberculosis, Chennai, TamilNadu India; 3 ICMR-National Institute of Epidemiology, Chennai, TamilNadu India

**Keywords:** Cytokines, Immunology, Monocytes and macrophages

## Abstract

Monocytes are thought to play an important role in host defence and pathogenesis of COVID-19. However, a comprehensive examination of monocyte numbers and function has not been performed longitudinally in acute and convalescent COVID-19. We examined the absolute counts of monocytes, the frequency of monocyte subsets, the plasma levels of monocyte activation markers using flowcytometry and ELISA in seven groups of COVID-19 individuals, classified based on days since RT-PCR confirmation of SARS-CoV2 infection. Our data shows that the absolute counts of total monocytes and the frequencies of intermediate and non-classical monocytes increases from Days 15–30 to Days 61–90 and plateau thereafter. In contrast, the frequency of classical monocytes decreases from Days 15–30 till Days 121–150. The plasma levels of sCD14, CRP, sCD163 and sTissue Factor (sTF)—all decrease from Days 15–30 till Days 151–180. COVID-19 patients with severe disease exhibit higher levels of monocyte counts and higher frequencies of classical monocytes and lower frequencies of intermediate and non-classical monocytes and elevated plasma levels of sCD14, CRP, sCD163 and sTF in comparison with mild disease. Thus, our study provides evidence of dynamic alterations in monocyte counts, subset frequencies and activation status in acute and convalescent COVID-19 individuals.

## Introduction

Monocytes plays a major role in the innate immune system and act as a vital component in the immune response to viral infections^1^. Monocytes are capable of recognizing, processing and presenting antigens to T cells and are also capable of producing cytokines and inflammatory markers that can regulate the immune response^2^. Based on surface expression markers—CD14 and CD16, monocytes are grouped into three populations: classical monocytes, intermediate monocytes and non-classical monocytes^3^. Each of these subsets is known to perform certain unique functions. The important role of monocytes in COVID-19 pathogenesis is emerging^4^.

Plasma levels of sCD14 and sCD163 have been established as strong indicators of monocyte activation^5^. Studies have shown that soluble tissue factor (sTF) secreted by cells of the monocyte/macrophage lineage plays an important role in the advancement and development of local and systemic inflammatory consequences^6,7^. C-reactive protein (CRP) is well known indicator of acute inflammation and its serum/plasma concentration is associated with the severity of systemic inflammation^8^. In HIV infection, monocyte activation markers like sCD14, sCD163, CRP and sTF have been extensively studied and shown to be associated with pathology^8–12^. Very few studies have examined the association of these monocyte activation markers (with the exception of CRP) in COVID-19. We set out to perform a detailed analysis of monocyte numbers, monocyte subset frequencies and circulating levels of monocyte activation markers in acute and convalescent COVID-19 individuals. Our study describes the dynamic alterations in monocyte counts, monocytes subsets and activation markers in acute and convalescent COVID-19 individuals up to more than 6 months following onset.

## Results

### Study population characteristics

The study population demographics and clinical characteristics are shown in Tables 1 and 2. There was no significant difference in age or sex between the study groups and other clinical parameters. The demographic and epidemiological data have been previously reported (Anuradha et al., American Journal of Tropical Medicine & Hygiene, 4th September 2021, AJTMH-21-0883).

**Table 1:** Demographics and clinical parameters of the study population.

Days after RT-PCR confirmation	15–30 days	31–60 days	61–90 days	91–120 days	121–150 days	151–180 days	More than 180 days
Subjects enrolled	n = 46	n = 33	n = 38	n = 34	n = 32	n = 37	n = 40
Median age (range)	41.5 (18–70)	36 (25–68)	45 (19–59)	45 (21–69)	45.5 (27–59)	42 (23–58)	38.5 (21–78)
Gender (M/F)	27/19	17/18	22/15	22/12	14/18	23/16	26/14
Fever, no. (%)	29 (67%)	22 (65%)	28 (74%)	23 (74%)	25 (83%)	23 (72%)	17 (47%)
Chills, no. (%)	9 (21%)	5 (15%)	2 (5%)	7 (22%)	4 (13%)	1 (3%)	3 (8%)
Cough, no. (%)	21 (49%)	20 (59%)	14 (37%)	15 (48%)	14 (47%)	17 (53%)	12 (33%)
Sore throat, no. (%)	21 (49%)	12 (35%)	11 (29%)	12 (38%)	10 (33%)	16 (50%)	13 (36%)
Runny nose, no. (%)	7 (16%)	6 (18%)	5 (13%)	0	3 (10%)	6 (19%)	5 (14%)
Taste loss, no. (%)	24 (55%)	14 (41%)	17 (44%)	12 (39%)	11 (37%)	20 (63%)	12 (33%)
Smell loss, no. (%)	21 (49%)	14 (41%)	21 (55%)	9 (29%)	11 (37%)	16 (50%)	10 (28%)
Muscle aches, no. (%)	23 (53%)	20 (59%)	29 (76%)	15 (48%)	18 (60%)	21 (66%)	13 (36%)
Joint pain, no. (%)	21 (49%)	18 (53%)	20 (53%)	10 (32%)	18 (60%)	14 (44%)	9 (25%)
Abdominal pain, no. (%)	3 (7%)	3 (9%)	4 (11%)	2 (6.5%)	3 (10%)	2 (7%)	3 (8%)
Vomit, no. (%)	3 (7%)	4 (12%)	5 (13%)	4 (13%)	3 (10%)	5 (16%)	3 (8%)
Diarrhea, no. (%)	10 (23%)	5 (15%)	4 (11%)	4 (13%)	6 (30%)	5 (16%)	2 (6%)
Seizures, no. (%)	0	1 (3%)	0	0	0	0	0
Hypertension, no. (%)	11 (26%)	7 (21%)	7 (18%)	7 (23%)	9 (30%)	9 (28%)	8 (22%)
Diabetes, no. (%)	8 (19%)	7 (21%)	11 (30%)	9 (29%)	11 (37%)	8 (25%)	7 (19%)
Asthma, no. (%)	2 (5%)	2 (6%)	1 (3%)	1 ( 3%)	0	1 (3%)	0
Chronic Kidney Disease, no. (%)	0	0	0	0	1 (3%)	0	1 (3%)
Neuro, no. (%)	0	0	2 (5%)	0	0	0	0
Cancer, no. (%)	0	0	0	0	0	0	0
Heart, no. (%)	1 (6%)	2 (3%)	1 (3%)	0	0	1 (3%)	0
Rheumatic fever, no. (%)	0	0	1 (3%)	0	0	1 (3%)	0
Corticosteroids, no. (%)	4 (9%)	3 ( 9%)	2 (5%)	3 (10%)	1 (3%)	1 (3%)	0
Antiviral drug, no. (%)	4 (9%)	5 (15%)	2 (5%)	4 (13%)	0	0	0
Immunomodulator, no. (%)	0	0	0	0	0	0	0

**Table 2:** Demographics and clinical parameters of the study population.

	Mild	Severe	*P* value
Subjects enrolled	n = 30	n = 15	
Median age (range)	32(18–69)	47 (22–61)	NS
Gender (M/F)	14/16	11/4	NS
Fever, no. (%)	25 (80.6%)	13 (86.7%)	NS
Chills, no. (%)	7 (22.6%)	3 (20%)	NS
Cough, no. (%)	14 (45.2%)	11 (73.3%)	NS
Sore throat, no. (%)	14 (45.2%)	4 (26.6%)	NS
Runny nose, no. (%)	6 (19.4%)	1 (6.7%)	NS
Taste loss, no. (%)	14 (45.2%)	8 (53.3%)	NS
Smell loss, no. (%)	16 (51.6%)	8 (53.3%)	NS
Muscle aches, no. (%)	22 (80%)	11 (73.3%)	NS
Joint pain, no. (%)	16 (51.6%)	9 (60%)	NS
Abdominal pain, no. (%)	5 (16.1%)	2 (13.3%)	NS
Vomiting, no. (%)	1 (3.2%)	2 (13.3%)	NS
Diarrhea, no. (%)	3 (9.7%)	6 (40%)	NS
Seizures, no. (%)	0	0	
Hypertension, no. (%)	5 (16.1%)	7 (46.7%)	NS
Diabetes, no. (%)	10 (32.3%)	8 (53.3%)	NS
Asthma, no. (%)	1 (3.2%)	0	
Chronic Kidney Disease, no. (%)	0	1	
Neuro, no. (%)	0	0	
Cancer, no. (%)	0	0	
Heart, no. (%)	1 (3.2%)	1(6.7%)	NS
Rheumatic fever, no. (%)	0	0	
Corticosteroids, no. (%)	0	3 (9%)	
Antiviral drug, no. (%)	1 (3.2%)	4 (15%)	NS
Immunomodulator, no. (%)	0	0	

### Increase in white blood cell (WBC) and monocyte absolute counts and percentages in convalescent COVID-19 individuals over time

**Figure 1:**
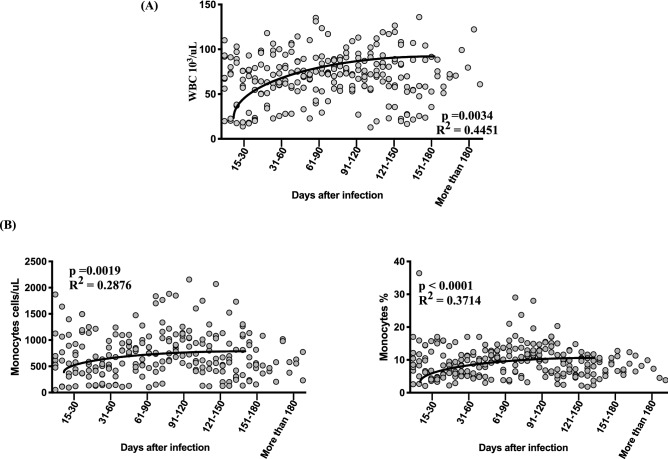
Increase in WBC and monocyte absolute counts and percentages in convalescent COVID-19 individuals over time. (**A**) Analysis of WBC absolute counts were shown using best fit curve model from acute and convalescent COVID-19 individuals classified as groups based on days since RT-PCR confirmation (**B**) Analysis of absolute monocyte counts and percentages were shown using best fit curve model from acute and convalescent COVID-19 individuals classified as groups based on days since RT-PCR confirmation. Analysis was done by first order model polynomial fit curve, for absolute count of WBC (p = 0.0034, R^2^ = 0.4451) and monocyte absolute count (percentage p = 0.0019, R^2^ = 0.2876 and p < 0.0001, R = 0.3714) by Akaike’s Information Criterion and each dot represent single individuals. Thick black line represents best fit curve.

To determine the absolute numbers of WBCs and absolute numbers and percentages of monocytes in acute and convalescent COVID-19 individuals over time, we examined them in the seven groups of COVID-19 individuals. As illustrated, absolute counts of WBC (Fig. 1A) and both absolute count and percentage of monocytes (Fig. 1B) were shown to increase from days 15–30 till 121–150 days following which the levels plateaued. Analysis was done by first order model polynominalfivt curve, for absolute count of WBC (R = 0.45) and monocyte absolute count (R = 0.37) by Akaike’s Information Criterion. Thus, both WBC and monocyte counts increase over time post COVID-19.

### Alterations in frequencies of circulating monocyte subsets in convalescent COVID-19 individuals over time

**Figure 2:**
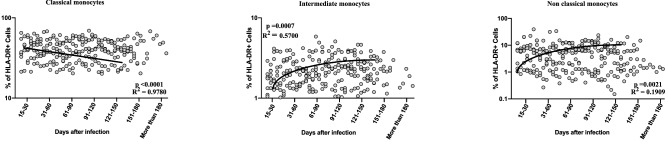
Alterations in frequencies of circulating monocyte subsets in convalescent COVID-19 individuals over time. Analysis of monocyte subsets from acute and convalescent COVID-19 individuals classified as groups based on days since RT-PCR confirmation. The frequencies of monocyte (classical, intermediate and non-classical) subsets are shown with first order model polynomial fit curve, classical monocyte p < 0.0001, R^2^ = 0.9780, intermediate monocytes p = 0.0007, R^2^ = 0.5700, non-classical monocytes p = 0.0021, R^2^ = 0.1909 by Akaike’s Information Criterion and each dot represent single individuals. Thick black line represents best fit curve.

To determine the frequencies and distribution of monocyte subsets in convalescent COVID-19 individuals over time, we assessed the *exvivo* frequencies of monocyte subsets (classical, intermediate and non-classical monocytes) in the seven groups of COVID-19 individuals. The gating method for the different monocyte subsets is illustrated in Supplementary Fig. 1. As shown in Fig. 2, cross sectional analysis exhibited that the frequencies of classical monocyte subsets started decreasing from days 15–30 (first order model polynominal fit curve, R = 0.41 by Akaike’s Information Criterion). After 151 days of infection, the frequencies of classical monocytes plateaued. In contrast, the frequencies of intermediate monocytes and non-classical monocytes started increasing from days 15–30 till 91–120 days (first order model polynominal fit curve, intermediate monocytes R = 0.34, non-classical monocytes R = 0.30 by Akaike’s Information Criterion). After 151 days, both the subsets plateaued. Thus, monocyte subset frequencies are altered over time post COVID-19.

### Increased levels of monocyte activation markers in convalescent COVID-19 individuals over time

**Figure 3:**
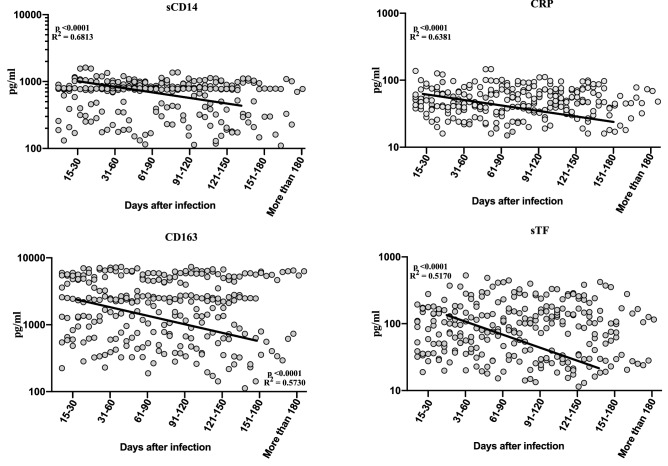
Increased levels of monocyte activation markers in convalescent COVID-19 individuals over time. Circulating plasma levels of monocyte activation markers sCD14, CRP, sCD163 and sTF from acute and convalescent COVID-19 individuals classified as groups based on days since RT-PCR confirmation. The levels of monocyte activation markers were shown with second order model polynomial fit curve, sCD14, p < 0.0001, R^2^ = 0.6813; CRP, p < 0.0001, R^2^ = 0.6381; sCD163, p < 0.0001, R^2^ = 0.5730, sTF, p < 0.0001, R^2^ = 0.5170 by Akaike’s Information Criterion and each dot represent single individuals. Thick black line represents best fit curve.

To determine the levels of monocyte activation markers in convalescent COVID-19 individuals over time, we assessed the plasma levels of sCD14, CRP, sCD163 and sTF in the seven groups of COVID-19 individuals. As shown in Fig. 3, cross sectional analysis demonstrated that the levels of sCD14, CRP, sCD163 and sTF started decreasing from days 15–30 (second order model polynominal fit curve, sCD14, R = 0.38, AICc = 201.2, Difference in AICc = − 12.82; CRP, R = 0.30, AICc = 227.3, Difference in AICc = − 6.32; sCD163, R = 0.37, AICc = 442.2, Difference in AICc-2.2 and sTF, R = 0.46, AICc = − 653, Difference in AICc-12.11 by Akaike’s Information Criterion) till 151 days after infection. Thus, plasma levels of monocyte activation markers are altered over time post COVID-19.

### Severe COVID-19 disease associated with altered frequencies of monocyte subsets and increased monocyte activation markers

**Figure 4:**
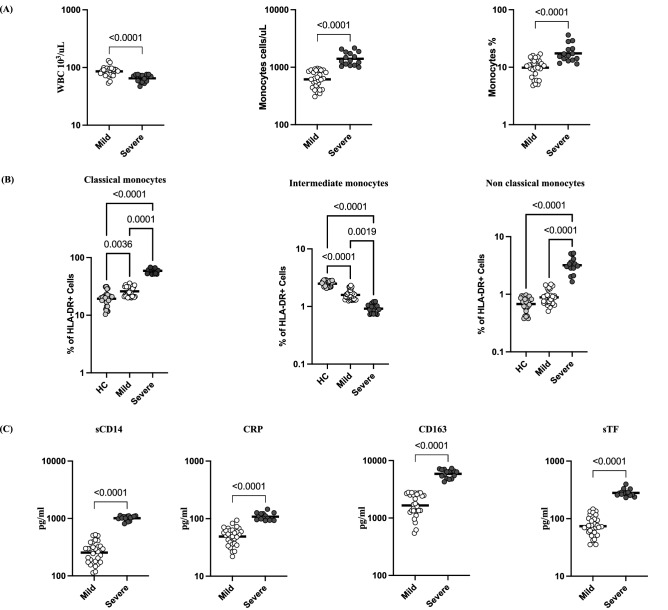
Severe COVID-19 disease associated with altered frequencies of monocyte subsets and increased monocyte activation markers. (**A**) WBC absolute count and Monocyte absolute count and percentage were shown for mild (n = 30) and severe (n = 15) COVID-19 individuals sampled between days 15 to 60 following RT-PCR confirmation. The data are represented as scatter plots with each circle representing a single individual. (**B**) The frequencies of monocyte subsets in mild (n = 30) and severe (n = 15) COVID-19 individuals sampled between days 15 to 60 following RT-PCR confirmation and healthy control individuals (n = 30). The data are represented as scatter plots with each circle representing a single individual. p values were calculated using the Kruskal–Wallis test. (**C**) Circulating plasma levels of monocyte activation markers sCD14, CRP, sCD163 and sTF in mild (n = 30) and severe (n = 15) COVID-19 sampled between days 15 to 60 following RT-PCR confirmation. The data are represented as scatter plots with each circle representing a single individual. p values were calculated using the Mann–Whitney U-test.

To examine the relationship between WBC and monocyte counts with disease severity, we determined the absolute counts of WBC and monocytes in COVID-19 individuals with mild and severe disease. As shown in Fig. 4A, the absolute count of WBC (Geomean (GM) of 85.5 × 10^3^/μl) in mild, 65 × 10^3^/μl) in severe, p < 0.0001) was significantly lower in severely diseased individuals. In contrast, monocyte absolute counts (GM of 615 cells/μl) in mild, 1405 cells/μl) in severe, p < 0.0001) and percentage of monocytes (GM of 9.8% in mild, 17.4% in severe, p < 0.0001) were significantly higher in severe COVID-19 compared with mild COVID-19.

Next, we determined the frequencies of monocyte subsets in mild and severely diseased COVID-19 individuals and compared them with healthy controls. As shown in Fig. 4B, the frequencies of classical (GM of 19% in HC, 26% in mild and 59% in severe, p < 0.0001) and non-classical monocytes (GM of 0.68% in HC, 0.88% in mild and 3.19% in severe, p < 0.0001) were significantly higher in severe compared with mild COVID-19 and healthy controls. In contrast, the frequencies of intermediate monocytes (GM of 2.5% in HC, 1.59% in mild and 0.92% in severe, p < 0.0001) were significantly lower in severe compared to mild COVID-19 and healthy controls. Further, we analysed the effect of COVID-19 disease severity on monocyte activation markers. As shown in Fig. 4C, the levels of sCD14 (GM of 254.3 pg/ml in mild, 1011 pg/ml in severe, p < 0.0001), CRP (GM of 48.9 pg/ml in mild, 108.2 pg/ml in severe, p < 0.0001), sCD163 (GM of 1653 pg/ml in mild, 5877 pg/ml in severe, p < 0.0001), and sTF (GM of 74 pg/ml in mild, 281 pg/ml in severe, p < 0.0001) were significantly higher in severe compared to mild COVID-19. Thus, severe COVID-19 disease is linked with altered frequencies of monocytes subsets and increased levels of monocyte activation markers.

## Discussion

While lymphopenia has been well described in COVID-19, the occurrence of monocytopenia is not well studied. Various studies have described decreased monocyte counts in severe disease^13–15^. This includes intermediate and non-classical monocytes in severe disease^16^, a decrease in only non-classical monocytes in severe disease^17,18^ and a marked decrease in non-classical monocytes in non-ICU patients compared to ICU patients^19^. A marked expansion of inflammatory monocytes has also been reported in COVID-19 patients and the percentage of CD14 ^+^ , CD16^ +^ monocytes secreting IL-6 correlates with disease severity^20,21^. However, very few studies have performed a long term analyses of changes in monocytes in convalescent COVID-19 individuals.

Our study is one of the first (to our knowledge) to perform a careful dissection of monocyte numbers, monocyte subset frequencies and plasma levels of monocyte activation markers in acute COVID-19 (defined as days 15–30 from detection) and different groups of convalescent COVID-19 (six groups characterized by duration from detection). We have classified convalescent COVID-19 individuals into different groups based on the duration from RT-PCR detection (which is the most accurate marker of infection) ranging from 31–60 days to longer than 180 days. Our data first demonstrate that acute COVID-19 individuals have diminished levels of absolute count of WBC as well as percentages and absolute counts of monocyte, which gradually increases following recovery from acute infection. Thus, our data reveals that the monocytopenia that is characteristic of acute COVID-19 is reversible and slowly returns back to normal levels around 4 to 6 months of post infection.

Our study also explores the dynamic alterations in monocyte subset frequencies at different time points following infection. Our data reveals that while classical monocytes show a trend towards gradual decline in frequencies until 4–6 months following infection, intermediate and non-classical monocytes shows a reverse trend with a gradual increase unto the same time point. Since classical monocytes are largely responsible for the production of pro-inflammatory cytokines during acute COVID-19, it stands to reason that classical monocytes have elevated frequencies in early infection with a substantial decline thereafter. Since non-classical and intermediate monocytes are mainly involved in immune surveillance and monitoring pathogen insults in the circulation, it is possible to speculate that the decrease in frequencies in early infection is directly related to acute infection and that the frequencies are gradually restored upon recovery from infection. Also, as non-classical and intermediate rarely undergo extravasation to tissues, it is unlikely that the decrease in frequencies can be attributed to migration into the lung in early infection.

Heightened levels of monocyte activation markers, such as sCD14, CRP, sCD163 and sTF have been associated with increased disease severity and increased risk of death in several viral infections^22,23^. CRP is one of the most reliable markers of disease severity and is a prognostic marker for increased morbidity and mortality in COVID-19^24,25^. sCD14 is a marker of immune pathology in COVID-19 and is a predictor of death in severe disease^26,27^. Similarly, sCD163 is also increased in COVID-19 patients^26^. sTF produced by monocytes in response to toll-ligands and cytokine signals can drive the initiation of the extrinsic coagulation cascade and promote increased levels of D-dimer and increased coagulation abnormalities^28^. Thus, these monocyte markers are all biomarkers of disease severity and pathogenesis in COVID-19. Our study clearly reveals that all the monocyte activation markers examined are elevated in acute COVID-19 and show a gradual decline with time. Thus, our data clearly demonstrates an association of elevated levels of monocyte activation markers with early infection and a diminution thereafter. More mechanistic studies detailing the role of sCD14, sCD163 and sTF in immune pathology of COVID-19 should be performed in the future.

Finally, our study also compared the absolute monocyte counts, percentages, subset frequencies and monocyte activation marker levels in mild versus severe COVID-19 individuals, whose samples were collected between Days 15 and 60 of RT-PCR confirmation. Our data clearly demonstrates that severe COVID-19 is associated with elevated monocyte counts, elevated frequencies of classical and non-classical monocytes and heightened plasma levels of monocyte activation markers. Thus, alterations in monocyte numbers and function appears to be a characteristic feature of severe COVID-19. Our study suffers from the limitation of not performing mechanistic studies on monocyte function. Nevertheless, our study provides a comprehensive examination of the evolution of monocyte activation and function over time in acute and convalescent COVID-19 individuals. Our study has the advantage of a fairly large sample size, a comprehensive set of data points including early infection to more than 6 months past infection and a thorough examination of monocyte numbers and subsets. Our study also provides the first detailed examination of activation marker levels in plasma of sCD14, sCD163 and sTF as well as their evolution over time. Our study thus implicates dynamic alterations in monocyte counts and function as one of the key events in COVID-19.

## Methods

### Study population

Acute COVID-19 (15–30 days from RT-PCR confirmation, n = 46) and Convalescent COVID-19 individuals (classified by days after infection as 31–60, n = 33; 61–90, n = 38; 91–120, n = 34; 121–150, n = 32; 151–180, n = 37 and more than 180, n = 40) and healthy control individuals (HC n = 30) residing in Chennai and Tiruvallur were recruited to the study between November 2020 and December 2020 after taking informed consent from the recruited individuals. Those who have active COVID-19 infection under home isolation and recovered COVID-19 patients within 0–15 days of RT-PCR confirmation were excluded from the study. The age group ranged between 18–75 years. COVID-19 was confirmed by RT-PCR in government approved laboratories. In brief, nasopharyngeal swabs, oropharyngeal (throat) swabs, were collected from persons who are doubted of COVID-19 by healthcare provider. RNA isolated and purified from specimens was reverse transcribed to cDNA and amplified. Thermocycling conditions consisted of 30 min at 48 °C for reverse transcription, 10 min at 95 °C for activation of the DNA polymerase, and 45 cycles of 15 s at 95 °C and 1 min at 60 °C. Fluorescence measurements were taken and the threshold cycle (CT) value for all sample was estimated by defining the point at which fluorescence surpassed a threshold limit set at the mean plus 10 standard deviations above the baseline. The results were positive if two or more of the SARS genomic targets exhibited positive results (CT < 45 cycles) and all positive and negative control reactions were in the expected range. Those individuals who did not experience any symptom during the entire course of illness were considered as asymptomatic and those who required supplemental oxygen support therapy or those who were admitted in ICU for oxygen support were considered as severely ill. Rest were classified under the mild illness category.

### Hematology and flow cytometry

Act-5 Diff hematology analyzer was used to measure the hematological parameters on all individuals (Beckman Coulter). Demographic details and other clinical parameters were shown in Table 1. Whole blood was used for ex vivo phenotyping and it was performed on all individuals. Briefly, 250 μl aliquots of whole blood was added to a cocktail of monoclonal antibodies. Monocyte phenotyping was performed using antibodies directed against CD45-PerCP (BD Biosciences), CD56 PerCP eFluor710 (eBiosciences), CD14-Pacific Blue (Biolegend), HLA-DR-PE-Cy7 (BD Biosciences) and CD16-APC-Cy7 (BD Biosciences). Classical monocytes were classified as CD45^+^ HLA-DR^+^ CD14^hi^ CD16^-^; intermediate monocytes as CD45 ^+^ HLA-DR ^+^ CD14^hi^ CD16^dim^ and non-classical monocytes were classified as CD45  HLA- DR ^+ ^CD14^dim^CD16^hi^. Followed by 30 min incubated at room temperature, 2 ml of FACS lysing solution (BD Biosciences Pharmingen) was used to lyse erythrocytes, the cells were washed 2 times with 2 ml of PBS and suspended in 200 ul of PBS (Lonza, Walkersville, MD). Eight- color flow cytometry was executed on a FACS Canto II flow cytometer with FACSDIVA software, version 6 (Becton Dickinson). The gating was set by forward and side scatter, and 1,00,000 gated events were acquired. Data were collected and analysed using FLOW JO software (TreeStar, Ashland, OR). Compensation and gating boundaries were adjusted using unstained, single stained, and Fluorescence Minus One (FMO) controls. FMO controls for each marker were used to calculate fluorescence intensity for the population. CD56 is the marker for NK cells. To exclude the NK cell populations and get pure monocyte populations, we gate on CD56- populations and further gated on HLA-DR and CD14 cells and then on CD16 and CD14.

### ELISA

Circulating plasma levels of sCD14, CRP, sCD163 and sTissue Factor were determined by Duoset ELISA kits (R & D systems) following manufacturer’s instructions. The detection limit as follows: sCD14- 62.5–4000 pg/ml; CRP- 15.6–1000 pg/ml; CD163- 156–10,000 pg/ml and sTissue Factor (sTF)- 7.8–500 pg/ml.

### Statistical analysis

Sample size calculation was conducted to obtain a power of 90% and Type I error of 0.05%. GraphPad PRISM version 9 (GraphPad Software, Inc., San Diego, CA, USA) was used for the data analyses. Cross-sectional analysis of frequency of monocyte subsets and hematology analysis was performed using polynomial model for best fit curve (either first order or second order model). Central tendency was measured by Geometric mean (GM). Statistically significant differences were calculated by nonparametric Mann–Whitney U test used for the comparison between mild versus severe.

### Ethics statement

The study was approved by the Ethics Committees of NIRT (NIRT-INo:2020047) and NIE (NIE/IHEC/202008-01).All the protocols were followed in consonance with the relevant institutional ethical committee guidelines.

## Supplementary Information

Supplementary Information.
