# Supplementary material for: Dynamic alterations in monocyte numbers, subset frequencies and activation markers in acute and convalescent COVID-19 individuals
Source: Sci Rep. 2021 Oct 12;11:20254. doi: 10.1038/s41598-021-99705-y (PMC8511073; doi:10.1038/s41598-021-99705-y)
Supplement: Supplementary file 1 — Supplementary Information. [file 41598_2021_99705_MOESM1_ESM.pdf]

Supplementary Figure.1.

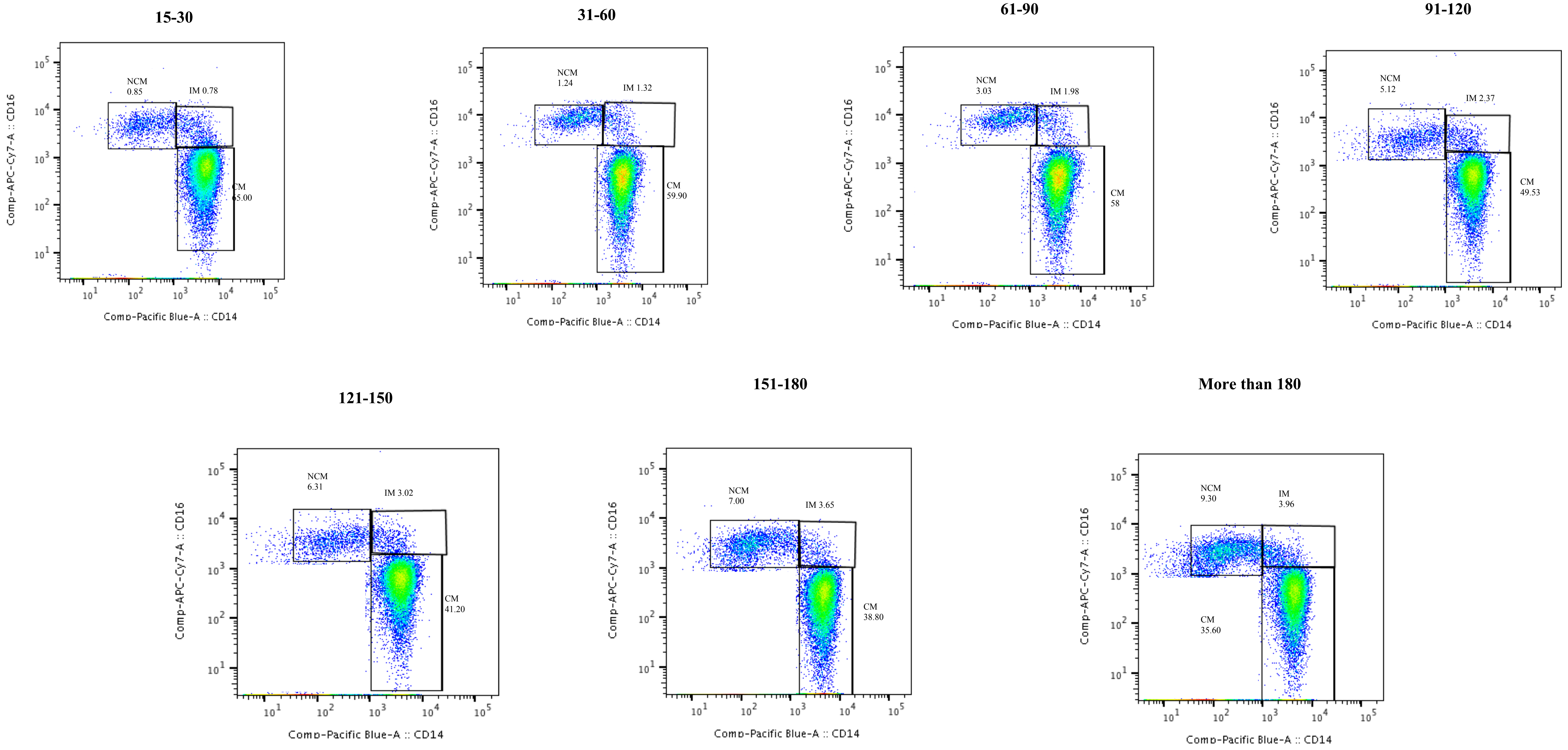

Supplementary Figure.1. A representative flow cytometry plot showing the gating strategy for estimation of monocyte subsets of 15-30, 31-60, 61-90, 91-120, 121-150, 151-180 and more than 180 days since RT-PCR confirmation of SARS-CoV2 infection.. Classical monocytes were classified as $CD45^{+}$ $HLA\text{-}DR^{+}$ $CD14^{hi}CD16^{-}$; intermediate monocytes as $CD45^{+}$ $HLA\text{-}DR^{+}$ $CD14^{hi}$ $CD16^{dim}$ and non-classical monocytes were classified as $CD45^{+}HLADR^{+}$ $CD14^{dim}CD16^{hi}$.
